# Effects of a multimodal intervention in primary care to reduce second line antibiotic prescriptions for urinary tract infections in women: parallel, cluster randomised, controlled trial 

**DOI:** 10.1136/bmj-2023-076305

**Published:** 2023-11-02

**Authors:** Guido Schmiemann, Alexandra Greser, Andy Maun, Jutta Bleidorn, Angela Schuster, Olga Miljukov, Viktoria Rücker, Anja Klingeberg, Anja Mentzel, Vitalii Minin, Tim Eckmanns, Christoph Heintze, Peter Heuschmann, Ildikó Gágyor

**Affiliations:** 1 University of Bremen, Department of Health Services Research, Institute for Public Health and Nursing Research, Bremen, Germany; 2 University Hospital Wurzburg, Department of General Practice, Wurzburg, Germany; 3 Institute of General Practice/Primary Care, Faculty of Medicine and Medical Center, University of Freiburg, Freiburg im Breisgau, Germany; 4 University Hospital Jena, Institute of General Practice, Jena, Thuringia, Germany; 5 Charité-Universitätsmedizin Berlin, Institute of General Practice and Family Medicine, Berlin, Germany; 6 Clinical Trial Centre Wurzburg, University Hospital Wurzburg, Germany; 7 Institute for Medical Data Sciences, University Hospital Wurzburg, Germany; 8 Julius-Maximilians-University of Wurzburg, Institute for Clinical Epidemiology and Biometry (ICE-B), Wurzburg, Germany; 9 Robert Koch-Institute, Berlin, Germany

## Abstract

**Objectives:**

To evaluate whether a multimodal intervention in general practice reduces the proportion of second line antibiotic prescriptions and the overall proportion of antibiotic prescriptions for uncomplicated urinary tract infections in women.

**Design:**

Parallel, cluster randomised, controlled trial.

**Setting:**

General practices in five regions in Germany. Data were collected between 1 April 2021 and 31 March 2022.

**Participants:**

General practitioners from 128 randomly assigned practices.

**Interventions:**

Multimodal intervention consisting of guideline recommendations for general practitioners and patients, provision of regional data for antibiotic resistance, and quarterly feedback, which included individual first line and second line proportions of antibiotic prescribing, benchmarking with regional or supra-regional practices, and telephone counselling. Participants in the control group received no information on the intervention.

**Main outcome measures:**

Primary outcome was the proportion of second line antibiotics prescribed by general practices, in relation to all antibiotics prescribed, for uncomplicated urinary tract infections after one year between the intervention and control group. General practices were randomly assigned in blocks (1:1), with a block size of four, into the intervention or control group using SAS version 9.4; randomisation was stratified by region. The secondary outcome was the prescription proportion of all antibiotics, relative within all cases (instances of UTI diagnosis), for the treatment of urinary tract infections after one year between the groups. Adverse events were assessed as exploratory outcomes.

**Results:**

110 practices with full datasets identified 10 323 cases during five quarters (ie, 15 months). The mean proportion of second line antibiotics prescribed was 0.19 (standard deviation 0.20) in the intervention group and 0.35 (0.25) in the control group after 12 months. After adjustment for preintervention proportions, the mean difference was −0.13 (95% confidence interval −0.21 to −0.06, P<0.001). The overall proportion of all antibiotic prescriptions for urinary tract infections over 12 months was 0.74 (standard deviation 0.22) in the intervention and 0.80 (0.15) in the control group with a mean difference of −0.08 (95% confidence interval −0.15 to −0.02, P<0.029). No differences were noted in the number of complications (ie, pyelonephritis, admission to hospital, or fever) between the groups.

**Conclusions:**

The multimodal intervention in general practice significantly reduced the proportion of second line antibiotics and all antibiotic prescriptions for uncomplicated urinary tract infections in women.

**Trial registration:**

German Clinical Trials Register (DRKS), DRKS00020389

## Introduction

Urinary tract infections (UTIs) in women lead to frequent consultation in primary care.^1^ Guidelines recommend symptomatic treatment and delayed prescription of antibiotics for women with mild to moderate UTI symptoms who wish to avoid antibiotics.^2^ ^3^ ^4^ However, in most primary care consultations antibiotics are still the first choice because they shorten the duration of symptoms and, to a lesser extent, these drugs also reduce the risk of complications such as pyelonephritis.^5^ ^6^ Implementing guideline recommendations on the basis of local resistance rates are core pillars of all antibiotic stewardship programmes, not only for urinary tract infections. Despite explicit recommendations for first line antibiotics, second line antibiotics such as fluoroquinolones still represent a large proportion of prescribed antibiotics for women with UTIs in Germany. Regional annual prescription rates of fluoroquinolones are between 38% and 54%^7^ and non-antibiotic treatments are rarely recommended by general practitioners.^8^

Various strategies to improve antibiotic prescribing behaviours (ie, to align to recommendations more closely) by healthcare providers in primary care have been explored but none has been identified as the most successful strategy.^9^ A Cochrane review of interventions targeted to the clinician to investigate the effect antibiotic prescribing in acute respiratory tract infections found that interventions such as shared decision making, educational materials, educational meetings, audit and feedback, and the use of point-of-care tests have an effect on prescribing behaviour.^10^ Multimodal interventions that comprise educational programmes and feedback about a physician’s prescribing behaviour have also shown positive effects on prescribing quality in ambulatory care.^11^ ^12^ ^13^ Based on the social norm theory, feedback of individual prescribing behaviour has been effective in reducing the rate of antibiotic prescriptions among clinicians who write high proportions of prescriptions compared with other clinicians.^14^ ^15^ Guidelines recommend and advocate for collecting information about regional resistance data in urinary tract infections but this recommendation is rarely implemented in practice.^3^ Although feedback on prescription quality has been used in intervention studies on UTIs, information about regional resistance data has not been applied to improve GPs’ adherence to guideline recommendations so far.^16^

Therefore, we aimed to investigate whether a multimodal intervention could reduce the number of prescriptions of second line antibiotics that GPs prescribe for women with UTIs. The intervention consisted of guideline recommendations for GPs and patients, provision of regional resistance data, and quarterly feedback that includes information on individual antibiotic prescribing proportions of first and second line antibiotics, benchmarking, and telephone counselling.

## Methods

### Trial design and setting

This cluster randomised controlled trial was conducted between 1 September 2019 and 31 December 2022. Data were collected between 1 April 2021 and 31 March 2022. Details of the study protocol have been published.^17^ General practices were the primary unit of randomisation and analysis. Data collected for the primary outcome were aggregated at practice level without access to individual patient data. Therefore, we did not need to account for classical clustering effects (eg, intra cluster correlation).

The primary target population were general practitioners in these regions in the south and east of Germany: Baden-Wurttemberg, Bavaria, Berlin, Brandenburg, and Thuringia. Funding requirements for this study did not align with the inclusion of private practices. Dedicated study teams enrolled participating practices using registers of affiliated practices, GP networks, and regional contacts.

### Randomisation and trial interventions

General practices were randomly assigned in blocks (1:1), with a block size of four, into the intervention or control group using SAS version 9.4; randomisation was stratified by region. Randomisation lists were generated by the Institute of Clinical Epidemiology and Biometry (University of Wurzburg).

The multimodal intervention consisted of guideline recommendations on UTI management for GPs and patients; provision of regional resistance data and feedback that included information about the proportions of individual first line and second line antibiotic prescriptions; benchmarking with regional (practices from the participating federal state) or supra-regional practices (from all participating federal states); and telephone counselling to discuss further questions. Feedback was offered on a quarterly basis (a quarter comprised three months).

In preparation for this project, data for regional resistance in uncomplicated urinary tract infections were collected in 136 primary care practices who did not participate in the randomised controlled trial. The resulting 2553 urine samples from women with uncomplicated UTIs were analysed and used to build the database to inform the intervention group on regional resistance data. As a result of expected relevant differences in resistance rates,^18^ regional resistance and susceptibility data were presented separately for UTI and for recurrent UTI (supplementary table 8). Neither the practice teams nor the research teams (one researcher and one research nurse in each region) were blinded to the intervention.

### Trial procedures

Study procedures were adapted to meet daily practice routine during a six month pilot phase in five non-participating practices in a region not participating in the trial. Additionally, acceptance and feasibility of the interventional procedures were assessed in two qualitative studies.^19^ ^20^ Piloting of the study led to minor adjustments in the information material and the composition and layout of the quarterly prescription feedback with no relevant changes in the intervention itself.^17^ Participants were recruited through practice networks within the respective regions (www.desam-fornet.de/en/). The recruitment phase lasted eight months from 1 July 2020 to 31 March 2021. After giving written informed consent, practices were randomly assigned a group and visited by the study teams (nurse or researcher, or both, adhering to covid-19 restrictions in place at the time). At the first visit, practices were informed about the study intervention in a face-to-face presentation and provided with the relevant information material. Because of covid-19 restrictions in some areas, this information was given via online presentation and intervention material was sent by mail. No further change in the study design was necessary. The impact of the pandemic on our trial is documented following the Conserve statement (supplementary material 2).^21^ The information material included a short (pocket card) and long version of the current guideline recommendations for the managements of UTI as issued by the national college of general practitioners and family physicians,^3^ ^4^ posters with information about the appropriate use according to the guideline of antibiotics, and patient brochures in five languages (German, English, Russian, Turkish, and Arabic). Resistance data and prescription feedback were sent out quarterly via email, mail, or fax as appropriate (supplementary table 8, supplementary figure 1). For further queries, telephone counselling provided by peers (senior researcher qualified as GP) was offered. All materials, including (video) instructions for data extraction, were also available via the study homepage (https://www.ukw.de/forschung/redares-projekt/startseite). Practices in the control group were informed about their allocation in a study investigating the treatment of urinary tract infections in women and the upcoming data extraction after 12 months (Q4). They were not aware of any of the components of the multimodal intervention. No further information about the study was published before our final data extraction to minimise contamination bias. The control group had no personal contact with the regional study team for 12 months (except at the last visit).

### Data collection

Electronic health record systems in Germany are diverse and not standardised, therefore, an automated extraction of patient files was not feasible. Instead, a medical practice assistant in each practice was trained by the research team to follow a detailed, standardised procedure for data extraction (supplementary material S1) to identify cases (instances of UTI diagnosis) and prescribed antibiotics from the electronic medical record. Data extraction was carried out at the end of each quarter over a 12 month period (Q1-Q4) in the intervention group and after 12 months in the control group. We documented first, second, and third antibiotic prescription for each instance of UTI. Information about non-antibiotic treatments such as painkiller, phytotherapy, or no documented treatment was also collected. Baseline data (Qb) was collected retrospectively using the data of the first quarter of 2020, one year before the study start. All data were verified by the relevant GPs. Data validation was performed once in the first quarter in each intervention practice. Research nurses randomly selected 10% of the UTI cases (three to five patients per practice) and compared all information in the patient file with the extracted data (supplementary material S2). Any inconsistency was clarified through personal contact with the medical practice assistants or, if required, with the GP.

All data were collected at practice level and transferred in an aggregated form each quarter to the coordinating study site. Data were extracted from the control practices 12 months after inclusion of the practice in the study. Data collection and analysis was unblinded.

Female patients with a documented uncomplicated UTI were identified via ICD 10 German modification-2020 code (N30.0, N30.9, N39.0, R30.0, R30.9). The GP’s clinical diagnosis of UTI was accepted in keeping with the pragmatic nature of the trial.^22^ Complicated UTI cases (ie, patients with flank pain, fever, or immune suppression) were excluded. In case of recurrent UTI, a woman could become a case more than once. According to current guidelines, we defined recurrent UTI as more than one infection in six months or more than two infections in 12 months.^2^ ^3^ We screened the electronic medical record for further diagnosis of a UTI up to 12 months before the diagnosis to detect recurrent UTI. Follow-up of individual patients with recurrent UTI was not feasible because data were aggregated at practice level. To detect complications such as pyelonephritis, fever, flank pain, urosepsis, and admission to hospital, patients’ electronic medical records were reviewed by the medical practice assistant until day 14 after the index diagnosis.

### Outcomes

The primary outcome was the proportion of second line antibiotics prescribed in relation to all antibiotics prescribed for uncomplicated UTIs after one year, calculated as the absolute difference in the mean proportion of the prescriptions between the control and intervention group. According to national guidelines, second line drugs were all antibiotics other than trimethoprim, pivmecillinam, nitrofurantoin, fosfomycin, or nitroxoline, which are first line treatments.^3^ ^4^

The secondary outcome was the proportion of all antibiotics prescribed for the treatment of UTIs after 12 months, calculated as the absolute difference in the mean proportion of all antibiotics prescribed between the intervention and control group. The prescription proportion of all antibiotics was defined as the proportion of first line and second line antibiotics within all UTI cases. Other exploratory outcomes were the proportion of high and low prescribers, the changes of prescribing behaviour over time and factors associated with low performance (defined as >10% of second line antibiotic prescriptions).

After publication of the study protocol, we added women with UTIs who were treated with any antibiotic after 12 months as an exploratory outcome to assess whether any changes could be observed with the intervention. For this outcome, we calculated the mean difference in the proportion of UTI cases treated with antibiotics in all UTI cases. Furthermore, we added cases with recurrent UTI and complications (eg, pyelonephritis, urosepsis, and hospital admissions) as exploratory outcomes.

### Data analysis

Data analysis followed the intention-to-treat principle on the full analysis set, which is as close as possible to an ideal intention-to-treat population. All randomly assigned practices remained in the allocated arm for analysis. Practices for which prescription data at baseline and from Q4 were available, formed the full analysis set. Prescription data were available and analysed as aggregated data for each practice and each quarter from Qb to Q4. Depending on the data type, descriptive statistics were used to summarise central tendencies (eg, means; variability, such as standard deviations; and frequencies of participant characteristics, prescribing patterns, and complications). For the primary and secondary hypotheses, analysis of covariance (ANCOVA) was used to adjust for the baseline proportion of prescribing and for region as defined a priori. Assumptions were examined before conducting ANCOVA to ensure the appropriateness of the statistical method.

Primary and secondary outcomes were treated as a continuous variable because the assumptions were met and we had aggregated data only. Complication rates were analysed as a nominal variable.

Like the exploratory outcomes, the secondary outcome was considered exploratory. Thus, no adjustment for multiple testing was made. Two sided tests such as t-test or χ^2^ test were used when assumptions were met, otherwise the non-parametric variant was used in bivariate analyses. We used negative binomial regression analysis (unadjusted and adjusted) to test for associations between predictors and proportions of second line antibiotic prescribing. All tests were done at a significance level of 5%.

The proportions of two practices in Qb and one practice in Q4 that had no prescribed antibiotics or any treatment in the intervention group were set to zero to keep them in the full analysis set for the primary and secondary analyses. In sensitivity analyses, primary and secondary outcomes were assessed after excluding these three practices. Further sensitivity analyses were conducted to evaluate the robustness of the means of the primary analysis including: (univariate) weighting by the inverse variance method; excluding practices with smaller numbers of diagnoses of UTIs (less than 5, 15, or 25); adjusting for region as well as for number of patients in a mixed effects meta-regression and applying multiple imputation that replaced missing data for prescription proportions in Qb or Q4. For the multiple imputation, missing data were assumed to be missing completely at random. A thousand iterations using predictive mean matching were done and effect estimates were pooled. Missing data in Qb, Q4, age, and years in practice were imputed by using information on these variables (if available) in addition to physician’s gender, practice location (region, community), average number of patients per quarter, and whether the practice was run by a GP with or without any partners. The data analyses were performed using R version 4.0.2.

### Sample size calculation

The sample size calculation for this study was based on our primary aim to reduce the proportion of second line antibiotics by 10 percent points per practice. To account for differing baseline prescribing proportions, the primary analysis used ANCOVA, with baseline prescribing proportions as the only covariate. Assumptions were made based on the findings of Dicheva and colleagues in 2015,^7^ stating that quinolone prescribing proportion lay at 0.43 of all antibiotics prescribed for women with a UTI. Data were not available at practice level; therefore, we assumed a standard deviation of 0.20 and moderate R^2^ of 0.25 of the covariate for the sample size calculation on the basis of clinical expert opinion obtained by discussion within the study team. Initially, a total sample size of 130 practices, 65 practices per group, was needed to detect an absolute difference of 0.10 in the prescribing proportion after 12 months between the control group (0.43) and the intervention group (0.33) with a power of 90% and a significance level of 5%. Assuming a dropout rate of 5% at the practice level, we aimed for a sample size of 138 practices. During the covid-19 pandemic, many practices were unable to participate due to their workload. However, we were able to recruit a total of 128 practices. Therefore, we performed a power analysis with the available practices and considered a higher dropout rate of 10% due to the impact of the pandemic. The results of the power analysis indicated that the study had a power of 86%, which was slightly lower than the initially planned power of 90%.

### Patient and public involvement 

We established a collaboration with a citizens’ forum consisting of 10 participants, established at the Department of General Practice, University of Wurzburg, Germany.^23^ Members of the forum were informed about the aims, processes, and materials for the study and were asked for their feedback, particularly regarding the patient brochure. As a result, minor language adaptations to improve understandability for non-specialists were made and the patient brochure was translated from German into English, Russian, Turkish, and Arabic.

An external study advisory board consisting of a GP, a pharmacist, a lay person, and a scientist with experience in this field was also involved to increase practice feasibility and to discuss the results. This advisory board did not lead to further changes in the study materials.

## Results

### Participants

**Fig 1:**
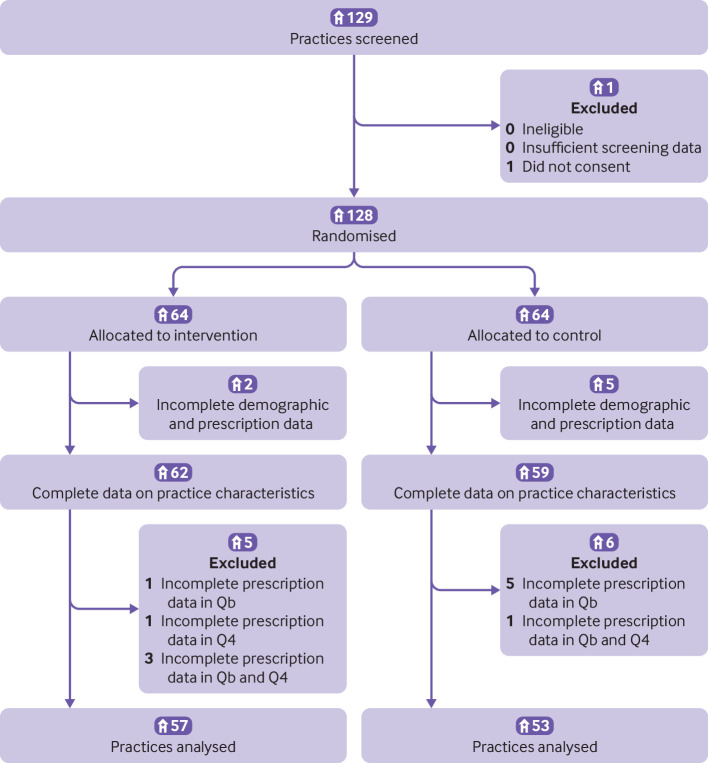
Flow diagram of participating practices throughout the trial. The final analysis included 57 practices in the intervention group and 53 practices in the control group. Q=quarter

On 1 April 2021, we randomly assigned 128 practices (64 practices to the intervention group and 64 to the control group), representing 203 GPs. Eleven practices in the intervention group did not extract data from Qb or Q4, or both, and seven did not extract data at all (fig 1). These practices, therefore, were not included in the full analysis set. Each practice comprised one to nine GPs. Characteristics of the practices and the participants were similar in both groups, with a small difference in practice region, but no differences in number of patients, gender, age group, individual physician’s work experience, or employment type (table 1, supplementary table 1).

**Table 1:** Baseline characteristics of the practices, number (percentage)

Characteristics	Intervention	Control	All
**Practice level**	**n=57**	**n=53**	**n=110**
Region:			
Berlin-Brandenburg	15 (26)	13 (25)	28 (25)
Baden-Württemberg	13 (23)	10 (19)	23 (21)
Bavaria	19 (33)	20 (38)	39 (35)
Thuringia	10 (18)	10 (19)	20 (18)
Mean no. of patients per quarter:			
500-999	14 (25)	9 (17)	23 (21)
1000-1499	24 (42)	18 (34)	42 (38)
1500 or more	19 (33)	26 (49)	45 (41)
Single practice	34 (60)	25 (47)	59 (54)
Rural community	9 (16)	11 (21)	20 (18)
No. of residents:			
Less than 5000	9 (16)	11 (21)	20 (18)
5000-20 000	23 (40)	19 (36)	42 (38)
>20 000-100 000	10 (18)	5 (9.4)	15 (14)
>100 000	5 (8.8)	13 (25)	18 (16)
>300 000	0 (0)	0 (0)	0 (0)
>500 000	10 (18)	5 (9.4)	15 (14)
**Participant level**	**n=103**	**n=100**	**n=203**
Self-reported gender			
Male	49 (48)	48 (48)	97 (48)
Female	54 (52)	52 (52)	106 (52)
Mean age (interquartile range), years:	50 (43-58)	53 (46-59)	52 (45-59)
(Missing)	2	3	5
Experience, years:			
≤5	8 (7.8)	4 (4.0)	12 (6.0)
6-15	35 (34)	24 (24)	59 (29)
≥15	59 (58)	71 (72)	130 (65)
(Missing)	1	1	2
Employment type:			
Full time	76 (75)	75 (75)	151 (75)
Part time	26 (25)	25 (25)	51 (25)
(Missing)	1	0	1
Position in practice:			
Owner	68 (67)	74 (75)	142 (71)
Employed doctor	34 (33)	25 (25)	59 (29)
(Missing)	1	1	2

### Outcomes

**Fig 2:**
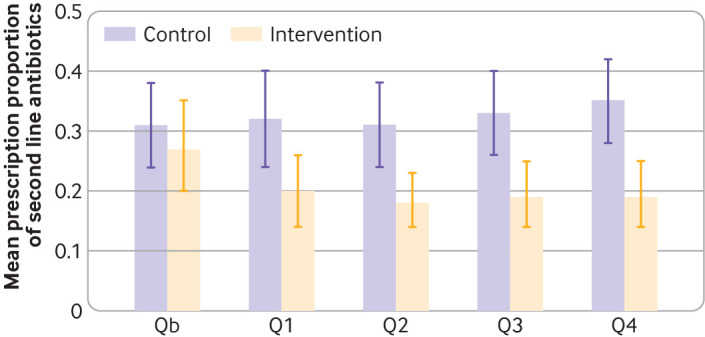
Proportions of second line antibiotic prescriptions in Qb-Q4. Percentiles: 10th (dashed line), 50th (solid line), and 90th percentile (dotted line). Q=quarter; Qb=baseline quarter

Overall, we identified 10 323 cases of UTIs from five quarters (ie, 15 months) in 110 practices included in the final analyses and to be analysed for the outcomes. The mean preintervention prescription proportions (Qb) for second line antibiotics in relation to all antibiotics for UTIs treatment were 0.27 (standard deviation 0.29) in the intervention group and 0.31 (0.25) in the control group (fig 2). The mean proportions of second line antibiotic prescriptions after 12 months were 0.19 (standard deviation 0.20) in the intervention group and 0.35 (0.25) in the control group. After adjustment for preintervention proportions, the mean difference was −0.13 (95% confidence interval −0.21 to −0.06, P<0.001), corresponding to a relative reduction of 40% (table 2).

**Table 2:** Primary, secondary, and exploratory outcomes in quarter four for the intervention group (n=57) and the control group (n=53)

Outcome	Unadjusted					Adjusted*		
	Intervention, mean (SD)	Control, mean (SD)	Difference† (95% CI)	P value†		Difference (95% CI)‡	P value‡	RR (95% CI)
Second line antibiotic prescription§	0.19 (0.20)	0.35 (0.25)	−0.15 (−0.24 to −0.06)	<0.001		−0.13 (−0.21 to −0.06)	<0.001	0.6 (0.31 to 0.89)
All antibiotic prescription¶	0.74 (0.22)	0.80 (0.15)	−0.06 (−0.14 to 0.01)	0.084		−0.08 (−0.15 to −0.01)	0.018	0.9 (0.81 to 0.98)
Urinary tract infection cases with any antibiotic prescription¶	0.72 (0.22)	0.77 (0.16)	−0.05 (−0.12 to 0.02)	0.19		−0.07 (−0.14 to 0.01)	0.068	0.91 (0.82 to 1.01)

CI=confidence interval; SD=standard deviation; RR=relative reduction (ratio of adjusted means).

* Adjusted for baseline prescribing proportions and region.

† Welch Two Sample t-test.

‡ ANCOVA (Analysis of Covariance).

§ Relevant within all antibiotic prescriptions.

¶ Relative within all cases (instances of urinary tract infection diagnosis).

The mean proportion of all antibiotic prescriptions for UTIs in relation to all UTI instances after 12 months was 0.74 (standard deviation 0.22) in the intervention and 0.80 (0.15) in the control group, with a mean difference of −0.08 (95% confidence interval −0.15 to −0.02, P<0.029; supplementary table 2). The ratio of adjusted means for the treatment group and control group was 0.90 (95% confidence interval 0.81 to 0.98), corresponding to a relative reduction of 10% (table 2). The proportion of second line antibiotics decreased from 0.27 to 0.20 in the intervention group when comparing the baseline with the first quarter after the intervention and this decline remained constant over the following quarters (fig 2; supplementary table 2 and 3). The mean proportion of cases treated with antibiotics was 0.72 (standard deviation 0.22) in the intervention group and 0.77 (0.16) in the control group after 12 months, with an adjusted mean difference of −0.07 (95% confidence interval −0.14 to 0.00, P=0.063).

**Fig 3:**
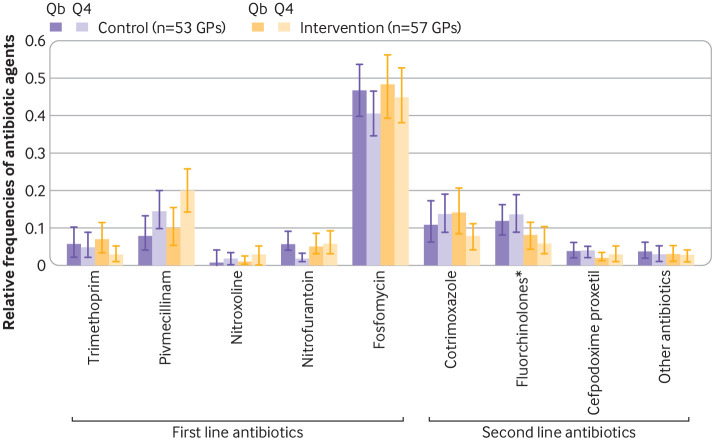
Relative frequencies of antibiotic agents in the control and intervention group in Qb and Q4. GPs=general practices; Q=quarter; Qb=baseline quarter*

Fosfomycin and pivmecillinam were the first line antibiotics most frequently used, while fluoroquinolones and trimethoprim-cotrimoxazole had the highest share in the group of second line antibiotics (fig 3).

Sensitivity analyses support the robustness of the primary results. When excluding the three intervention practices without prescriptions in the quarters Qb and Q4, the estimates remained stable (supplementary table 4). When weighting the means by the number of cases in the practices, the reduction of the second line antibiotics remained stable (supplementary table 5). This pattern was also present in practices that had at least 25 diagnoses of UTI in Qb and Q4, in which more precise proportion estimates were available. The results also remained consistent when applying the meta regression, accounting for baseline prescribing, region, and the number of UTI diagnoses within the same model (supplementary table 6). The assumptions for ANCOVA were checked (supplementary figure 2) and one outlier was detected during the assumption checks of the ANCOVA but this datapoint did not alter the results relevantly when excluded. After multiple imputation for 11 practices with missing values in Q4 or Qb, or both, results were similar to the original data (adjusted mean difference 0.13, P<0.001).

### Complication rates

The rate of complications (ie hospital admissions, recurrent UTI, fever, pyelonephritis, flank pain, or urosepsis) in 12 months, documented within 14 days after the initial diagnosis, was very low overall (table 3). Pyelonephritis was documented in 0.2% in both groups, however, flank pain, a possible clinical sign for pyelonephritis, was documented in 0.7% of all cases in the intervention group versus 1.1% in the control group. The rate of recurrent UTI was lower in the intervention group compared with the control group (12% *v* 17%).

**Table 3:** Complications in all cases of urinary tract infections

Characteristics	Baseline quarter (Qb)					Intervention period (Q1-Q4)			
	Intervention, n (%) (n=884)	Control, n (%) (n=1064)	P value*	All, n (%) (n=1948)		Intervention, n (%) (n=4115)	Control, n (%) (n=4262)	P value†	All, n (%) (n=8377)
Admission to hospital	3 (0.3)	5 (0.5)	0.74	8 (0.4)		9 (0.2)	10 (0.2)	0.88	19 (0.2)
Recurrent urinary tract infection	128 (14)	154 (14)	>0.99	282 (14)		475 (12)	741 (17)	<0.001	1216 (15)
Fever	2 (0.2)	7 (0.7)	0.20	9 (0.5)		11 (0.3)	19 (0.4)	0.17	30 (0.4)
Pyelonephritis	1 (0.1)	0 (0)	0.45	1 (<0.1)		7 (0.2)	8 (0.2)	0.85	15 (0.2)
Flank pain	15 (1.7)	22 (2.1)	0.55	37 (1.9)		30 (0.7)	45 (1.1)	0.11	75 (0.9)
Urosepsis	0 (0)	0 (0)	—	0 (0)		1 (<0.1)	3 (<0.1)	—	4 (<0.1)

Q=quarter.

* Fisher's exact test; Pearson's χ^2^ test.

† Pearson's χ^2^ test.

### High and low prescribing practices

**Fig 4:**
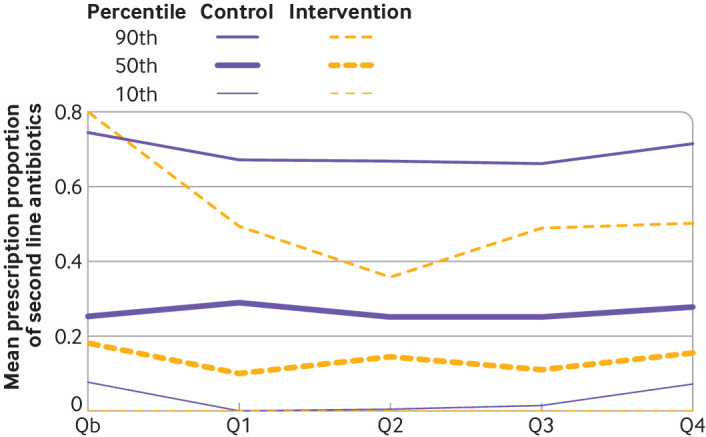
Mean prescription proportions of second line antibiotics by quarter

Comparing high and low prescribing practices of second line antibiotics, we found a marked decrease of practices that were deemed to be prescribing a high amount (90th percentile) in the intervention group without similar changes in the control group (fig 4). This effect could not be shown among GPs who prescribe an average amount (50th percentile) or a low amount (10th percentile) where differences between the groups remained stable.

In the negative binomial regression (supplementary table 7), we sought to explain the pattern of prescribing of second line antibiotics. The model shows that single doctor practices are more likely to prescribe second line antibiotics compared with joint practices at an incidence rate ratio of 1.48 (95% confidence interval 1.05 to 2.08, P=0.026).

## Discussion

### Principal findings

The multimodal intervention consisting of guideline recommendations for GPs and patients, provision of regional resistance data, and quarterly feedback of individual antibiotic prescribing proportions, benchmarking, and telephone counselling resulted in a decrease in prescription proportions of second line antibiotics for uncomplicated UTIs in women. Additionally the intervention group noted a reduction of all antibiotics prescribed for this indication, with no evidence for an increase in complications.

Disease specific quality indicators for outpatient antibiotic prescribing in Europe recommend a prescription rate of less than 5% for quinolones in adult women who were diagnosed with cystitis.^24^ This threshold was nearly reached in our intervention group (6%). Overall, the prescription proportions for second line antibiotics at the beginning of the study were lower than we had assumed based on routinely collected data from 2012 to 2013.^7^ More recent data for prescribing frequencies in Germany has documented a marked decline in the prescription of fluoroquinolones decreasing from 29.4% to 8.7% between 2015 and 2019.^25^ One explanation might be that the covid-19 pandemic reduced attention to a more restricted use of fluorquinolones as recommended by the European Medical Agency,^26^ thus enhancing the effect of the intervention. Our results also appear relevant more broadly in Europe, where quinolone prescription rates vary from 3% in Sweden to 22% in Belgium.^27^ Belgium has reported large reductions because of changes in reimbursement criteria.^28^

Our study showed a reduction of antibiotic prescription proportions and an increase of UTI cases treated without antibiotics, which is in line with the recommendation of many guidelines.^2^ ^3^ More than 10 years ago, antibiotic prescription rates between 80% and 100% for women with UTIs were proposed as a quality indicator.^24^ Due to new evidence on non-antibiotic treatment in women with uncomplicated UTI,^29^ this indicator is not valid any more. However, no consensus exists yet on the optimal rate of antibiotic prescriptions. Qualitative and survey data suggest that many women are open to try non-antibiotic treatments as a first treatment choice.^1^ ^30^ The rate of women treated with antibiotics for UTIs, however, differs between countries. A comparison between Belgium, Netherlands, and Sweden found rates between 67% and 87%.^27^

### Comparison with other studies

The intervention was found to be sustained over the one year period and was most effective in practices with high proportions of second line prescriptions. The results are in line with Schwartz and colleagues who were able to show a reduction in antibiotic prescription rates for respiratory tract infections in high prescribing GPs using a single letter.^14^ Targeting future interventions to high prescribers only might be the most effective approach because we could show the greatest benefit in this group. To achieve better results, peer discussion rounds on educational material and prescription rates could be promoted and incentivised in, for example, existing quality circles. Similar measures were successfully implemented by the Swedish strategic programme for the rational use of antimicrobial agents and surveillance of resistance (STRAMA).^13^ When aiming to reduce antibiotic prescription in general practice, no gold standard exists. In several well designed randomised controlled trials with a complex intervention, different changes of antibiotics prescriptions behaviour were observed.^31^ ^32^ Aghlmandi and colleagues aimed to improve prescription rates in respiratory and urinary tract infections by audit and feedback with peer benchmarking, similar to our study but without a positive effect.^31^ Using a multifaceted complex intervention with interactive, multimedia, and electronic components Vellinga and colleagues could increase the rate of first line prescribing in UTI by 25% accompanied by an unintended increase in overall antimicrobial prescribing for UTI.^32^ The study by Hartman and colleagues reduced the number of antibiotic prescriptions for suspected urinary tract infections in frail older adults (mean age of 86 years) by an absolute 50% but a comparison is difficult because the intervention was focused on an algorithm guided diagnosis in older people, which is a different topic in a different population.^13^ Which component of the multifaceted intervention has the highest impact is unclear. Providing data for regional resistance rates is a new approach. These data are required by national and international guidelines and necessary because in routine practice, resistance rates from uncomplicated UTI are unknown because available data are biased by complicated cases.^18^ Additionally, data showed regional differences in resistance to first line and second line antibiotics, allowing adjustment of treatment choices. Furthermore, data inform guidelines for treatment recommendations. Kurotschka and colleagues showed that resistance of *Escherichia* *coli* gradually decreased over time after fluoroquinolone use declined.^33^ Similar results were shown in previous studies.^34^ ^35^ Thus, a positive effect on resistance rates of *E coli* can be expected.

### Complications

Despite a lower proportion of antibiotic prescriptions in the intervention group, complication rates within the 12 month period were similar in both groups. The rate of admissions to hospital in our study (0.2%) was identical to the results of a nationwide, register cohort study in Sweden including 752 289 women with acute uncomplicated UTIs.^36^ By contrast, our rate of pyelonephritis (0.2%) was lower than the 1% observed in the Swedish cohort study.^36^ One explanation might be the different length of the follow up (three months *v* two weeks in our study). Another explanation is the documentation of flank pain in the free text of the patients’ electronic medical records, which showed 0.7% prevalence (1.1% control group) in our study. This indication might suggest pyelonephritis but evidence was probably insufficient for a definitive diagnosis.

Our exploratory analysis showed differences in the rates of recurrent UTIs (table 3). Increased use of second line antibiotics was associated with higher resistance rates of *E coli* and treatment failure.^37^ Since, in our study, the resistance rates for recurrent UTI were in favour of first line antibiotics due to their low resistance rates, this could explain the reduced rates of recurrent infections in the intervention group. However, we cannot rule out a documentation bias because the time between training for and conducting data extraction for recurrent UTI was different in the two groups.

### Strengths and limitations

The main strength of our study comprises data for regional resistance rates as a component of the multimodal intervention that has not been used in intervention studies to improve prescribing patterns in UTI before.^16^ Additionally, the resistance data provided distinguished between first time and recurrent UTIs (supplementary table 8), which allowed for a more individualised clinical approach and was highly appreciated by participants.^19^

Our rigorous data extraction allowed us to focus on uncomplicated UTIs by using additional information from the electronic medical record to exclude complicating factors such as fever, flank pain, or immunosuppression at inclusion. This method is an advantage over routinely collected data that does not allow for accurate identification of uncomplicated UTIs. However, implementation of the project on a larger scale is limited because of the resources needed.

During the intervention phase, 18 (14%) of 128 practices could not be included in the full analysis set because of insufficient or no data extraction. The most frequent reason for this was the high workload during the pandemic.

The lack of blinding in the practice teams may have affected the validity of the results. Reporting bias and a contamination bias were possible because intervention and control practices were located in the same area. We believe the reporting bias was minimised by use of an objective endpoint, training of medical practice assistants, and validation of data by the study team. Contamination could have influenced prescribing behaviour. The prescription proportions in the control group, however, argue against a substantial impact from no blinding because this would have tended to reduce the effect size of the intervention. To mitigate the risk of systematic errors in data extraction, research nurses randomly checked data in the practices. Nevertheless, we cannot rule out an observer bias due to awareness of the intervention. Blinding of the statistician was also not possible at the analysis stage, because the regular analysis of the data was necessary to implement components of the interventions, for example, for the feedback analysis described. However, all statistical analyses were agreed on in advance in a detailed statistical analysis plan. Moreover suggestions from current practice and guidelines of blinding statisticians in clinical trials varies widely and is uncertain.^38^ Therefore, we believe that the risk of bias due to an unblinded statistician is likely to be low in our study.

We acknowledge the reliance on expert opinion for the estimation of the standard deviation as a limitation of our study. Yet, the standard deviation observed in our study was found to be consistent with the estimated standard deviation, suggesting that the expert opinion was reasonably aligned with the actual variability in the prescription proportions among the participating practices. This alignment indicates that the estimated standard deviation accurately captured the dispersion of the data and supports the validity of the power calculation.

Another limitation is that we could not identify delayed prescriptions because these were not routinely documented in the electronic medical record. Therefore, the proportion of non-antibiotic treatment may be different than reported because delayed prescriptions are an accepted treatment option for women with uncomplicated UTI.^39^ Possible solutions could be incentives for documentation by GPs and an automated data extraction tool that also allows analysis of free text in the electronic patient records^40^ or access to digitalised patient files, including information on retrieval of drugs from pharmacies.

We were not able to consider the number of patients in the practice and could not determine a denominator for our analyses because no fixed patient lists are available in German general practices and the number of patients varies from quarter to quarter. Numbers are likely to have varied at random in both groups of practices. Therefore, we could not gain information about whether consultation rates changed with the intervention or not.

### Implications for practice

The study could show that the multifaceted intervention works and we assume that all components can be easily implemented in countries where the level of digitalisation allows an automated data extraction and feedback is possible. In Germany, implementing of the materials is possible for example within the continuous postgraduate medical education or work in quality circles. We do not expect relevant barriers in implementing resistance data but they have to be collected and presented at regional level because they are affected by different antibiotic recommendations or policies.

After our study showed high resistance rates in women with recurrent UTIs, the current update of the German guidelines for the treatment of uncomplicated UTIs, which has not yet been published, will recommend against trimethoprim in women with recurrent UTI.

### Conclusions

The multimodal intervention comprising the provision of guideline recommendations, information about regional resistance data, and individualised feedback on antibiotic prescription proportions, increased GPs’ guideline adherence and reduced antibiotic prescribing in women with uncomplicated UTI in German general practices. If implemented on a larger scale, our results are likely to have a sustainable positive impact on antibiotic stewardship programmes for uncomplicated UTI in primary care.

**What is already known on this topic**

In uncomplicated urinary tract infections, symptomatic (non-antibiotic) treatment is an option that is recommended by current guidelines
Despite explicit recommendations, second line antibiotics are still often used in uncomplicated urinary tract infections
Interventions including educational programmes and prescribing feedback have shown a reduction of inappropriate prescribing, encouraging a non-antibiotic treatment has not been addressed by intervention programmes so far

**What this study adds**

A multimodal intervention including guideline information, individual prescribing feedback, and provision of regional resistance data might reduce the proportion of second line antibiotics and antibiotic prescriptions in uncomplicated urinary tract infections

## Data Availability

The datasets (anonymised aggregated data at practice level) used and analysed during this study will be available from the corresponding author on reasonable request.

## Web extra

Extra material supplied by authors

Web appendix: Online appendix

## Notes

Contributors: IG, GS, AM, CH, and JB conceived the idea of the study. AG, AK, TE, AS, AME, and VM acquired data and supervised data collection in the practices. AG, AK, TE, AS, AME, and VM were involved in the statistical concept and analysis. All authors analysed and interpreted the data. IG and GS drafted the manuscript. The guarantor (IG) accepts full responsibility for the work and the conduct of the study, had access to the data, and controlled the decision to publish. The corresponding author (AG) attests that all listed authors meet authorship criteria and that no others meeting the criteria have been omitted.

Patient consent: Informed consent was only obtained from the physicians; patient informed consent was not needed because only aggregated and anonymised data were collected.

The lead author (the manuscript's guarantor IG) affirms that the manuscript is an honest, accurate, and transparent account of the study being reported; that no important aspects of the study have been omitted; and that any discrepancies from the study as originally planned (and, if relevant, registered) have been explained.

Dissemination to participants and related patient and public communities: Study results will be presented to all participating practices and all information developed (patient information in various languages, posters, data on resistance rates) including the study homepage will be accessible to the public. Resistance rates are already included in the current update of the national guideline on urinary tract infections. The material developed will also be included in continuous medical education programmes.

Provenance and peer review: Not commissioned; externally peer reviewed.

## References

[1] Butler CC Hawking MKD Quigley A McNulty CAM . Incidence, severity, help seeking, and management of uncomplicated urinary tract infection: a population-based survey. Br J Gen Pract 2015;65:e702-7. 10.3399/bjgp15X686965 26412847 PMC4582883

[2] Scottish Intercollegiate Guidelines Network (SIGN) . Management of suspected bacterial lower urinary tract infection in adult women. 2020. https://www.sign.ac.uk/our-guidelines/management-of-suspected-bacterial-lower-urinary-tract-infection-in-adult-women/

[3] Kranz J Schmidt S Lebert C Schneidewind L Schmiemann G Wagenlehner F . Uncomplicated bacterial community-acquired urinary tract infection in adults. Dtsch Arztebl Int 2017;114:866-73. 10.3238/arztebl.2017.0866 29271346 PMC5763001

[4] Schmiemann G Gebhardt K Hummers E . Burning on micturition (Brennen beim Wasserlassen) guideline of the German College of General Practitioner and Family Physicians DEGAM, 2018. https://www.degam.de/degam-leitlinien-379

[5] Pouwels KB Dolk FCK Smith DRM Robotham JV Smieszek T . Actual versus ‘ideal’ antibiotic prescribing for common conditions in English primary care. J Antimicrob Chemother 2018;73(suppl_2):19-26. 10.1093/jac/dkx502. 29490060 PMC5890776

[6] Ong Lopez AMC Tan CJL Yabon AS 2nd Masbang AN . Symptomatic treatment (using NSAIDS) versus antibiotics in uncomplicated lower urinary tract infection: a meta-analysis and systematic review of randomized controlled trials. BMC Infect Dis 2021;21:619. 10.1186/s12879-021-06323-0 34187385 PMC8243445

[7] Dicheva S . Harnwegsinfekte bei Frauen [Urinary tract infections in women]. Barmer GEK Arzneimittel report 2015. 1st edn. Siegburg: Asgard Verlagsservice, 2016. https://www.barmer.de/resource/blob/1026444/60143006d7108440f02512a6a80fcaea/barmer-gek-arzneimittel-report-2015-data.pdf

[8] Gágyor I Strube-Plaschke S Rentzsch K Himmel W . Management of urinary tract infections: what do doctors recommend and patients do? An observational study in German primary care. BMC Infect Dis 2020;20:813. 10.1186/s12879-020-05377-w. 33167875 PMC7650164

[9] Arnold SR Straus SE . Interventions to improve antibiotic prescribing practices in ambulatory care. Cochrane Database Syst Rev 2005;2005:CD003539. 10.1002/14651858.CD003539.pub2. 16235325 PMC7003679

[10] Tonkin-Crine SK Tan PS van Hecke O . Clinician-targeted interventions to influence antibiotic prescribing behaviour for acute respiratory infections in primary care: an overview of systematic reviews. Cochrane Database Syst Rev 2017;9:CD012252. 10.1002/14651858.CD012252.pub2. 28881002 PMC6483738

[11] Butler CC Simpson SA Dunstan F . Effectiveness of multifaceted educational programme to reduce antibiotic dispensing in primary care: practice based randomised controlled trial. BMJ 2012;344:d8173. 10.1136/bmj.d8173. 22302780 PMC3270575

[12] Vervloet M Meulepas MA Cals JWL Eimers M van der Hoek LS van Dijk L . Reducing antibiotic prescriptions for respiratory tract infections in family practice: results of a cluster randomized controlled trial evaluating a multifaceted peer-group-based intervention. NPJ Prim Care Respir Med 2016;26:15083. 10.1038/npjpcrm.2015.83. 26845640 PMC4741286

[13] Hartman EAR van de Pol AC Heltveit-Olsen SR . Effect of a multifaceted antibiotic stewardship intervention to improve antibiotic prescribing for suspected urinary tract infections in frail older adults (ImpresU): pragmatic cluster randomised controlled trial in four European countries. BMJ 2023;380:e072319. 10.1136/bmj-2022-072319. 36813284 PMC9943914

[14] Schwartz KL Ivers N Langford BJ . Effect of antibiotic-prescribing feedback to high-volume primary care physicians on number of antibiotic prescriptions: a randomized clinical trial. JAMA Intern Med 2021;181:1165-73. 10.1001/jamainternmed.2021.2790. 34228086 PMC8261687

[15] Hallsworth M Chadborn T Sallis A . Provision of social norm feedback to high prescribers of antibiotics in general practice: a pragmatic national randomised controlled trial. Lancet 2016;387:1743-52. 10.1016/S0140-6736(16)00215-4. 26898856 PMC4842844

[16] Cox S Lo-A-Foe K van Hoof M . Physician-targeted interventions in antibiotic prescribing for urinary tract infections in general practice: a systematic review. Antibiotics (Basel) 2022;11:1560. 10.3390/antibiotics11111560. 36358215 PMC9686805

[17] Gágyor I Greser A Heuschmann P . REDuction of Antibiotic RESistance (REDARES) in urinary tract infections using treatments according to national clinical guidelines: study protocol for a pragmatic randomized controlled trial with a multimodal intervention in primary care. BMC Infect Dis 2021;21:990. 10.1186/s12879-021-06660-0. 34556027 PMC8461906

[18] Klingeberg A Noll I Willrich N . Antibiotic-resistant E. coli in uncomplicated community-acquired urinary tract infection. Dtsch Arztebl Int 2018;115:494-500. 10.3238/arztebl.2018.0494. 30135009 PMC6121086

[19] Petruschke I Stichling K Greser A Gagyor I Bleidorn J . [The general practitioner perspective of a multimodal intervention for the adequate use of antibiotics in urinary tract infection - a qualitative interview study]. Z Evid Fortbild Qual Gesundhwes 2022;170:1-6. 10.1016/j.zefq.2021.12.012. 35283054

[20] Mentzel A Maun A . Outpatient antibiotic prescription behavior and attitudes towards prescription feedback. Z Allg Med 2023;99:21-7. 10.1007/s44266-022-00007-x.

[21] Orkin AM Gill PJ Ghersi D Campbell L Sugarman J Emsley R . Guidelines for reporting trial protocols and completed trials modified due to the covid-19 pandemic and other extenuating circumstances: the CONSERVE 2021 statement. JAMA 2021;326:257-65. 34152382 10.1001/jama.2021.9941

[22] Loudon K Treweek S Sullivan F Donnan P Thorpe KE Zwarenstein M . The PRECIS-2 tool: designing trials that are fit for purpose. BMJ 2015;350:h2147. 10.1136/bmj.h2147 25956159

[23] Schilling I Behrens H Bleidorn J . Patients’ and researchers’ experiences with a patient board for a clinical trial on urinary tract infections. Res Involv Engagem 2019;5:38. 10.1186/s40900-019-0172-0. 31798964 PMC6882213

[24] Adriaenssens N Coenen S Tonkin-Crine S Verheij TJ Little P Goossens H The ESAC Project Group . European Surveillance of Antimicrobial Consumption (ESAC): disease-specific quality indicators for outpatient antibiotic prescribing. BMJ Qual Saf 2011;20:764-72. 10.1136/bmjqs.2010.049049. 21441602

[25] Schmiemann G Hoffmann F Hamprecht A Jobski K . Patterns and trends of antibacterial treatment in patients with urinary tract infections, 2015-2019: an analysis of health insurance data. BMC Prim Care 2022;23:204. 10.1186/s12875-022-01816-6 35948891 PMC9367112

[26] European Medicines Agency. 2018. Quinolone- and fluoroquinolone-containing medicinal products. https://www.ema.europa.eu/en/medicines/human/referrals/quinolone-fluoroquinolone-containing-medicinal-products (accessed 13.07.2023)

[27] Tyrstrup M van der Velden A Engstrom S . Antibiotic prescribing in relation to diagnoses and consultation rates in Belgium, the Netherlands and Sweden: use of European quality indicators. Scand J Prim Health Care 2017;35:10-8. 10.1080/02813432.2017.1288680. 28277045 PMC5361413

[28] Vermeulen H Coenen S Hens N Bruyndonckx R . Impact of changing reimbursement criteria on the use of fluoroquinolones in Belgium. J Antimicrob Chemother 2021;76:2725-32. 10.1093/jac/dkab255 34374778 PMC8446932

[29] Kaußner Y Röver C Heinz J . Reducing antibiotic use in uncomplicated urinary tract infections in adult women: a systematic review and individual participant data meta-analysis. Clin Microbiol Infect 2022;28:1558-66. 10.1016/j.cmi.2022.06.017. 35788049

[30] Gbinigie OA Tonkin-Crine S Butler CC Heneghan CJ Boylan AM . Non-antibiotic treatment of acute urinary tract infection in primary care: a qualitative study. Br J Gen Pract 2022;72:e252-60. 10.3399/BJGP.2021.0603. 35314431 PMC8966781

[31] Aghlmandi S Halbeisen FS Saccilotto R . Effect of antibiotic prescription audit and feedback on antibiotic prescribing in primary care: a randomized clinical trial. JAMA Intern Med 2023;183:213-20. 10.1001/jamainternmed.2022.6529. 36745412 PMC9989898

[32] Vellinga A Galvin S Duane S . Intervention to improve the quality of antimicrobial prescribing for urinary tract infection: a cluster randomized trial. CMAJ 2016;188:108-15. 10.1503/cmaj.150601 26573754 PMC4732960

[33] Kurotschka PK Fulgenzio C Da Cas R . Effect of fluoroquinolone use in primary care on the development and gradual decay of *Escherichia coli* resistance to fluoroquinolones: a matched case-control study. Antibiotics (Basel) 2022;11:822. 10.3390/antibiotics11060822 35740228 PMC9219874

[34] Bakhit M Hoffmann T Scott AM Beller E Rathbone J Del Mar C . Resistance decay in individuals after antibiotic exposure in primary care: a systematic review and meta-analysis. BMC Med 2018;16:126. 10.1186/s12916-018-1109-4 30081902 PMC6091205

[35] Costelloe C Metcalfe C Lovering A Mant D Hay AD . Effect of antibiotic prescribing in primary care on antimicrobial resistance in individual patients: systematic review and meta-analysis. BMJ 2010;340:c2096. 10.1136/bmj.c2096 20483949

[36] Jansåker F Li X Vik I Frimodt-Møller N Knudsen JD Sundquist K . The risk of pyelonephritis following uncomplicated cystitis: a nationwide primary healthcare study. Antibiotics (Basel) 2022;11:1695. 10.3390/antibiotics11121695. 36551352 PMC9774091

[37] Fasugba O Gardner A Mitchell BG Mnatzaganian G . Ciprofloxacin resistance in community- and hospital-acquired Escherichia coli urinary tract infections: a systematic review and meta-analysis of observational studies. BMC Infect Dis 2015;15:545. 10.1186/s12879-015-1282-4. 26607324 PMC4660780

[38] Iflaifel M Partlett C Bell J . Blinding of study statisticians in clinical trials: a qualitative study in UK clinical trials units. Trials 2022;23:535. 10.1186/s13063-022-06481-9 35761345 PMC9235168

[39] Duane S Beatty P Murphy AW Vellinga A . Exploring experiences of delayed prescribing and symptomatic treatment for urinary tract infections among general practitioners and patients in ambulatory care: a qualitative study. Antibiotics (Basel) 2016;5:27. 10.3390/antibiotics5030027. 27537922 PMC5039523

[40] Holte M Holmen J . Program for data extraction in primary health records: a valid tool for knowledge production in general practice? BMC Res Notes 2020;13:23. 10.1186/s13104-020-4887-7. 31924277 PMC6954497

