# Supplementary material for: Effects of a multimodal intervention in primary care to reduce second line antibiotic prescriptions for urinary tract infections in women: parallel, cluster randomised, controlled trial
Source: BMJ. 2023 Nov 2;383:e076305. doi: 10.1136/bmj-2023-076305 (PMC10620739; doi:10.1136/bmj-2023-076305)
Supplement: Supplementary file 1 — Web appendix: Online appendix [file schg076305.ww.pdf]

Data Supplement to

Reduction in prescribing second-line antibiotics for urinary tract infections: a cluster randomised controlled trial by a multimodal intervention in primary care

Supplementary table 1

Characteristics	Respondents, N = 110[1]	Non-Respondents, N = 11[1]	p-value[2]	All, N = 121[1]
Region			0.030	
Berlin	28 (25)	2 (18)		30 (25)
BaWü	23 (21)	7 (64)		30 (25)
Bavaria	39 (35)	2 (18)		41 (34)
Thuringia	20 (18)	0 (0)		20 (17)
Average number of patients per quartal according to practice			0.7	
1.000 or less	23 (21)	1 (9.1)		24 (20)
More than 1.000	87 (79)	10 (91)		97 (80)
Average number of patients per quartal according to practice			0.2	
500 - 1.000	23 (21)	1 (9.1)		24 (20)
1.000 - 1.500	42 (38)	2 (18)		44 (36)
1.500 or more	45 (41)	8 (73)		53 (44)
Single Practice	59 (54)	9 (82)	0.11	68 (56)
Rural community	20 (18)	2 (18)	>0.9	22 (18)
Number of Residents			>0.9	
Less than 5.000	20 (18)	2 (18)		22 (18)
5.000 - 20.000	42 (38)	4 (36)		46 (38)
20.000 - 100.000	15 (14)	2 (18)		17 (14)
> 100.000	18 (16)	2 (18)		20 (17)
> 300.000	0 (0)	0 (0)		0 (0)
> 500.000	15 (14)	1 (9.1)		16 (13)
	Respondents, N = 203[1]	Non-Respondents, N = 18[1]	p-value[2]	All, N = 221[1]
**Participant level**				
Gender			0.12	
m	97 (48%)	12 (67%)		109 (49%)
w	106 (52%)	6 (33%)		112 (51%)
Age in Years (= 2021-Birthyear)	51 (10)	51 (13)	>0.9	51 (10)
(Missing)	5	0		5

Characteristics	Respondents, N = 110[1]	Non-Respondents, N = 11[1]	p-value[2]	All, N = 121[1]
Experience in years			0.6	
<= 5 Years	12 (6.0%)	1 (5.6%)		13 (5.9%)
6-15 Years	59 (29%)	7 (39%)		66 (30%)
>= 15 Years	130 (65%)	10 (56%)		140 (64%)
(Missing)	2	0		2
Employment Type			0.8	
Full-time	151 (75%)	13 (72%)		164 (75%)
Part-time	51 (25%)	5 (28%)		56 (25%)
(Missing)	1	0		1
Position in Practice			0.7	
Owner	142 (71%)	12 (67%)		154 (70%)
Employed Doctor	59 (29%)	6 (33%)		65 (30%)
(Missing)	2	0		2

[1]n (%); Mean (SD)

[2]Pearson's Chi-squared test; Fisher's exact test; Welch Two Sample t-test; Wilcoxon rank sum test

Supplementary table 2
Absolute and relative antibiotic prescriptions in Control and Intervention group at practice level from Qb to Q4

	Qb[3]			Q1[4]		Q2[4]		Q3[4]		Q4[4]			Total (Qb to Q4)[5]		
**Characteristics**	**Intervention, N = 57[2]**	**Control, N = 53[2]**	**p-value[1]**	**Intervention, N = 57[2]**	**Control, N = 53[2]**	**Intervention, N = 57[2]**	**Control, N = 53[2]**	**Intervention, N = 57[2]**	**Control, N = 53[2]**	**Intervention, N = 57[2]**	**Control, N = 53[2]**	**p-value[1]**	**Intervention, N = 57[2]**	**Control, N = 53[2]**	**All, N = 110[2]**
UTI Cases			0.054									0.23			
Mean (SD)	16 (11)	20 (13)		16 (9)	18 (13)	20 (12)	22 (14)	19 (12)	21 (13)	17 (12)	20 (14)		18 (11)	20 (13)	19 (12)
Range	3, 56	3, 63		1, 41	1, 61	5, 60	0, 61	3, 59	3, 69	0, 53	3, 57		0, 60	0, 69	0, 69
Sum	884	1,064		905	935	1,136	1,169	1,087	1,089	984	1,070		4,996	5,327	10,323
Second-line antibiotic prescriptions (absolute)			0.24									<0.001			
Mean (SD)	3.8 (5.4)	4.9 (4.8)		2.5 (3.4)	4.7 (5.5)	2.9 (3.3)	5.9 (6.3)	3.0 (4.6)	5.5 (5.4)	2.6 (3.1)	5.3 (4.4)		3.0 (4.1)	5.3 (5.3)	4.1 (4.8)
Sum	216.0	261.0		141.0	250.0	166.0	312.0	172.0	289.0	149.0	280.0		844.0	1,392.0	2,236.0
All antibiotic prescriptions (absolute)			0.16									0.11			
Mean (SD)	13 (10)	16 (11)		13 (9)	15 (11)	16 (11)	19 (13)	15 (11)	18 (12)	14 (10)	17 (11)		14 (10)	17 (12)	15 (11)
Sum	766	867		718	793	904	1,008	874	932	770	890		4,032	4,490	8,522
UTI cases with any antibiotic prescription (absolute)			0.17									0.19			
Mean (SD)	12 (9)	15 (10)		12 (7)	14 (10)	14 (10)	17 (11)	14 (10)	16 (11)	13 (9)	15 (10)		13 (9)	15 (10)	14 (10)
Sum	696	780		657	724	819	901	801	857	716	795		3,689	4,057	7,746
Second-line antibiotic prescriptions (relative within			0.45									<0.001			

	Qb[3]			Q1[4]		Q2[4]		Q3[4]		Q4[4]			Total (Qb to Q4)[5]		
**Characteristics**	**Intervention, N = 57[2]**	**Control, N = 53[2]**	**p-value[1]**	**Intervention, N = 57[2]**	**Control, N = 53[2]**	**Intervention, N = 57[2]**	**Control, N = 53[2]**	**Intervention, N = 57[2]**	**Control, N = 53[2]**	**Intervention, N = 57[2]**	**Control, N = 53[2]**	**p-value[1]**	**Intervention, N = 57[2]**	**Control, N = 53[2]**	**All, N = 110[2]**
all antibiotic prescriptions)															
Mean (SD)	0.27 (0.29)	0.31 (0.25)		0.20 (0.22)	0.32 (0.28)	0.18 (0.18)	0.31 (0.25)	0.19 (0.21)	0.33 (0.25)	0.19 (0.20)	0.35 (0.25)		0.21 (0.22)	0.32 (0.26)	0.26 (0.25)
All antibiotic prescriptions (relative within all antibiotic,non-antibiotic and no prescriptions)			0.26									0.084			
Mean (SD)	0.82 (0.18)	0.79 (0.16)		0.79 (0.22)	0.81 (0.19)	0.78 (0.18)	0.80 (0.19)	0.77 (0.20)	0.83 (0.14)	0.74 (0.22)	0.80 (0.15)		0.78 (0.20)	0.81 (0.17)	0.79 (0.19)
UTI cases with any antibiotic prescription (relative)			0.17									0.19			
Mean (SD)	0.79 (0.19)	0.74 (0.17)		0.73 (0.21)	0.77 (0.18)	0.73 (0.18)	0.76 (0.19)	0.73 (0.20)	0.80 (0.15)	0.72 (0.22)	0.77 (0.16)		0.74 (0.20)	0.77 (0.17)	0.75 (0.19)

[1]Welch Two Sample t-test

[2]Mean (SD = Standard Deviation); Range; Sum

[3] Baseline quarter

[4] Intervention period

[5]The total number (Qb to Q4) is the average frequency of prescribed antibiotics per practices over the quarters

Supplementary table 3

Antibiotic agent prescriptions in Control and Intervention group at practice level from Qb to Q4 (relative within all antibiotic prescriptions)

	Qb[2]		Q1[3]		Q2[3]		Q3[3]		Q4[3]	
**Characteristics**	**Intervention, N = 57[1]**	**Control, N = 53[1]**	**Intervention, N = 57[1]**	**Control, N = 53[1]**	**Intervention, N = 57[1]**	**Control, N = 53[1]**	**Intervention, N = 57[1]**	**Control, N = 53[1]**	**Intervention, N = 57[1]**	**Control, N = 53[1]**
Antibiotic agent					First-line					
Trimethoprim	0.07 (0.15)	0.06 (0.14)		0.04 (0.08)	0.05 (0.12)	0.05 (0.12)	0.05 (0.12)	0.04 (0.08)	0.03 (0.09)	0.05 (0.13)
Pivmecillinam	0.10 (0.20)	0.08 (0.16)	0.17 (0.23)	0.13 (0.19)	0.18 (0.20)	0.16 (0.20)	0.18 (0.22)	0.14 (0.20)	0.20 (0.24)	0.15 (0.19)
Nitroxoline	0.01 (0.05)	0.01 (0.07)	0.00 (0.02)	0.00 (0.01)	0.00 (0.03)	0.00 (0.02)	0.02 (0.10)	0.01 (0.03)	0.03 (0.10)	0.02 (0.05)
Nitrofurantoin	0.05 (0.10)	0.06 (0.10)	0.06 (0.13)	0.05 (0.09)	0.05 (0.07)	0.02 (0.05)	0.07 (0.11)	0.03 (0.06)	0.06 (0.11)	0.02 (0.04)
Fosfomycin	0.48 (0.31)	0.47 (0.26)	0.50 (0.29)	0.43 (0.25)	0.53 (0.29)	0.44 (0.23)	0.46 (0.27)	0.45 (0.22)	0.45 (0.28)	0.41 (0.22)
					Second-line					
Cotrimoxazole	0.14 (0.22)	0.11 (0.21)	0.08 (0.15)	0.13 (0.21)	0.07 (0.13)	0.10 (0.13)	0.08 (0.14)	0.13 (0.21)	0.08 (0.14)	0.14 (0.18)
Fluorchinolones	0.08 (0.12)	0.12 (0.15)	0.07 (0.15)	0.13 (0.20)	0.04 (0.09)	0.13 (0.20)	0.05 (0.11)	0.11 (0.17)	0.06 (0.12)	0.14 (0.18)
Cefpodoxime proxetil	0.02 (0.05)	0.04 (0.08)	0.02 (0.04)	0.02 (0.05)	0.04 (0.07)	0.04 (0.07)	0.04 (0.09)	0.04 (0.11)	0.03 (0.08)	0.04 (0.05)
Other antibiotic agent	0.03 (0.08)	0.04 (0.07)	0.03 (0.06)	0.04 (0.07)	0.03 (0.06)	0.04 (0.08)	0.02 (0.05)	0.05 (0.10)	0.03 (0.05)	0.03 (0.07)

[1]Mean (SD = Standard Deviation)

[2] Baseline quarter

[3] Intervention period

Sensitivity Analyses

Supplementary table 4

Primary outcome (without the three intervention practices without prescriptions in the quarters Qb and Q4)

	Unadjusted					Adjusted[5]		
**Outcome**	**Intervention, N = 54[1]**	**Control, N = 53[1]**	**Difference[2]**	**95% CI[2, 3]**	**p-value[2]**	**Difference[4]**	**95% CI[3, 4]**	**p-value[4]**
Second-line antibiotic prescriptions in Q4 (relative within all antibiotic prescriptions)	0.21 (0.20)	0.35 (0.25)	-0.14	-0.23 to -0.05	0.002	-0.12	-0.19 to -0.04	0.002

[1]Mean (Standard Deviation = SD)
[2]Welch Two Sample t-test
[3]CI = Confidence Interval
[4]ANCOVA
[5]Adjusted for baseline prescribing proportions and region

Supplementary table 5

Weighted Means for second-line antibiotic prescription proportions per quarter

Second-line antibiotic prescription proportions per quarter

	Qb[1]		Q1[2]		Q2[2]		Q3[2]		Q4[2]	
Group	Mean (sd)	Weighted mean (sd)*	Mean (sd)	Weighted mean (sd)*	Mean (sd)	Weighted mean (sd)*	Mean (sd)	Weighted mean (sd)*	Mean (sd)	Weighted mean (sd)*
Control	0.313 (0.245)	0.219 (0.273)	0.322 (0.273)	0.228 (0.343)	0.307 (0.251)	0.226 (0.316)	0.329 (0.252)	0.250 (0.329)	0.346 (0.253)	0.263 (0.302)
Intervention	0.275 (0.287)	0.203 (0.337)	0.201 (0.218)	0.050 (0.114)	0.185 (0.176)	0.060 (0.106)	0.196 (0.205)	0.050 (0.108)	0.198 (0.193)	0.046 (0.099)

*Weighted means and standard deviations are calculated by the inverse variance method using the practice's number of treatment cases as sample sizes to determine the variances

[1] Baseline quarter

[2] Intervention period

Weighted Means for practices with minimum number of cases per quarter

Second-line antibiotic prescription proportions with practices with at least 5 cases per quarter

Group	Qb[1]		Q1[2]		Q2[2]		Q3[2]		Q4[2]	
	Mean (sd)	Weighted mean (sd)*	Mean (sd)	Weighted mean (sd)*	Mean (sd)	Weighted mean (sd)*	Mean (sd)	Weighted mean (sd)*	Mean (sd)	Weighted mean (sd)*
Control	0.319 (0.244)	0.225 (0.274)	0.327 (0.256)	0.218 (0.329)	0.314 (0.253)	0.226 (0.316)	0.327 (0.256)	0.250 (0.330)	0.344 (0.247)	0.272 (0.303)
Intervention	0.277 (0.273)	0.195 (0.325)	0.192 (0.194)	0.047 (0.098)	0.185 (0.176)	0.060 (0.106)	0.199 (0.205)	0.051 (0.109)	0.207 (0.191)	0.047 (0.100)

*Weighted means and standard deviations are calculated by the inverse variance method using the practice's number of treatment cases as sample sizes to determine the variances

[1] Baseline quarter

[2] Intervention period

Weighted Means for practices with medium number of cases per quarter

Second-line antibiotic prescription proportions with practices with at least 15 cases per quarter

Group	Qb[1]		Q1[2]		Q2[2]		Q3[2]		Q4[2]	
	Mean (sd)	Weighted mean (sd)*	Mean (sd)	Weighted mean (sd)*	Mean (sd)	Weighted mean (sd)*	Mean (sd)	Weighted mean (sd)*	Mean (sd)	Weighted mean (sd)*
Control	0.312 (0.208)	0.227 (0.199)	0.300 (0.220)	0.152 (0.198)	0.297 (0.200)	0.172 (0.192)	0.308 (0.195)	0.287 (0.248)	0.325 (0.211)	0.214 (0.198)
Intervention	0.287 (0.253)	0.201 (0.247)	0.179 (0.196)	0.050 (0.103)	0.173 (0.144)	0.064 (0.091)	0.166 (0.170)	0.049 (0.098)	0.224 (0.197)	0.060 (0.112)

*Weighted means and standard deviations are calculated by the inverse variance method using the practice's number of treatment cases as sample sizes to determine the variances

[1] Baseline quarter

[2] Intervention period

Weighted Means for practices with maximum number of cases per quarter

Second-line antibiotic prescription proportions with practices with at least 25 cases per quarter

	Qb[1]		Q1[2]		Q2[2]		Q3[2]		Q4[2]	
Group	Mean (sd)	Weighted mean (sd)*	Mean (sd)	Weighted mean (sd)*	Mean (sd)	Weighted mean (sd)*	Mean (sd)	Weighted mean (sd)*	Mean (sd)	Weighted mean (sd)*
Control	0.280 (0.196)	0.198 (0.186)	0.289 (0.170)	0.175 (0.172)	0.306 (0.203)	0.197 (0.196)	0.305 (0.217)	0.292 (0.268)	0.299 (0.225)	0.184 (0.191)
Intervention	0.278 (0.234)	0.208 (0.255)	0.205 (0.222)	0.053 (0.109)	0.161 (0.178)	0.063 (0.090)	0.187 (0.195)	0.064 (0.118)	0.146 (0.121)	0.047 (0.071)

*Weighted means and standard deviations are calculated by the inverse variance method using the practice's number of treatment cases as sample sizes to determine the variances

[1] Baseline quarter

[2] Intervention period

Supplementary table 6

Primary Outcome Mixed-Effects Meta-Regression Models

**Outcome**	**Difference**	**95% CI[1,2]**	**p-value**
Second-line antibiotic prescriptions in Q4 (relative within all antibiotic prescriptions) adjusted for case size	-0.15	-0.22 to -0.08	<0.001
Second-line antibiotic prescriptions in Q4 (relative within all antibiotic prescriptions) adjusted for region and case size	-0.15	-0.22 to -0.07	<0.001

[1]CI = Confidence Interval

[2] Knapp and Hartung method

Supplementary table 7

Negative binomial regression model for the association of predictors with second-line prescribing

	Unadjusted			Adjusted					
**Variable**	**IRR[1]**	**95% CI[1]**	**p-value**	**UTI cases N**	**Event N**	**Proportion**	**IRR[1]**	**95% CI[1]**	**p-value**
**Single Practice**			**0.039**						**0.026**
No	1.00	—		1,123	197	18%	1.00	—	
Yes	1.44	1.02 - 2.03		825	199	24%	1.48	1.05 - 2.08	
**Teaching pratice**			0.10						0.087
No	1.00	—		1,240	284	23%	1.00	—	
Yes	0.73	0.50 - 1.06		708	112	16%	0.73	0.51 - 1.05	
**Rural Community**			0.26						0.082
No	1.00	—		1,537	329	21%	1.00	—	
Yes	0.77	0.49 - 1.22		411	67	16%	0.67	0.43 - 1.05	
**(Intercept)**				1,948	396	20%	0.20	0.15 - 0.27	**<0.001**
Null deviance							124		
Null default							109		
Deviance							115		
Residual default							106		

[1]IRR = Incidence Rate Ratio, CI = Confidence Interval

Supplementary table 8

Handout of current resistance rates for first-and second-line antibiotics (per region) for GPs

RedAres

ROBERT KOCH INSTITUT

Current resistance shares of recommended antibiotics for the calculated therapy of community-acquired uncomplicated cystitis (southeastern region)

	Antibiotic/ antibiotic class	Uncomplicated UTI			
		Non-recurrent UTI		Recurrent UTI	
		Resistance share	95% KI	Resistance share	95% KI
1st line ab*	**Fosfomycin**	**2,2 %**	**0,8 - 6,3**	**1,0 %**	**0,2 - 5,6**
	**Nitrofurantoin**	**0,0 %**	**0,0 - 1,9**	**0,7 %**	**0,1 - 3,9**
	**Nitroxolin**	**0,5 %**	**0,1 - 3,0**	**0,7 %**	**0,1 - 4,1**
	**Pivmecillinam**	**4,0 %**	**2,0 - 7,7**	**3,5 %**	**1,5 - 8,0**
	**Trimethoprim**	**14,6 %**	**10,4 - 20,2**	**18,4 %**	**12,9 – 25,6**
2nd line ab *	Cefpodoxim	4,5 %	2,0 - 10,2	3,4 %	1,2 - 9,7
	Ciprofloxacin	5,0 %	2,7 - 8,9	14,9 %	10,0 - 21,7
	Cotrimoxazol	10,9 %	7,3 - 16,0	16,3 %	11,1 – 23,3
	Levofloxacin	7,6 %	3,7 - 14,9	18,5 %	10,4 - 30,8
	Norfloxacin	8,9 %	4,6 - 16,6	18,5 %	10,4 - 30,8
	Ofloxacin	NA	–	NA	–

- Antibiotic recommended (Resistance rate <5%)
- Antibiotic recommended (Resistance rate ≥5 - <15 %)
- Antibiotic recommended (Resistance rate 15 - 20 %)
- Antibiotic recommended as second choice (Resistance rate <5%)
- Antibiotic recommended as second choice (Resistance rate ≥5 - <15 %)
- Antibiotic conditionally recommended as second choice (resistance rate 15 - 20 %)
- Antibiotic not recommended (resistance rate ≥20 %)
- Antibiotic not recommended (resistance rate ≥20 %)
- NA No data

*According to the guideline programme DGU: Interdisziplinäre S3 Leitlinie: Epidemiologie, Diagnostik, Therapie, Prävention und Management unkomplizierter, bakterieller, ambulant erworbener Harnwegsinfektionen bei erwachsenen Patienten. Langversion 1.1-2, 2017 AWMF Registernummer: 043/044

Supplementary Figure 1

Feedback and benchmarking handout with prescription proportions of first-and second-line antibiotics per quarter for GPs

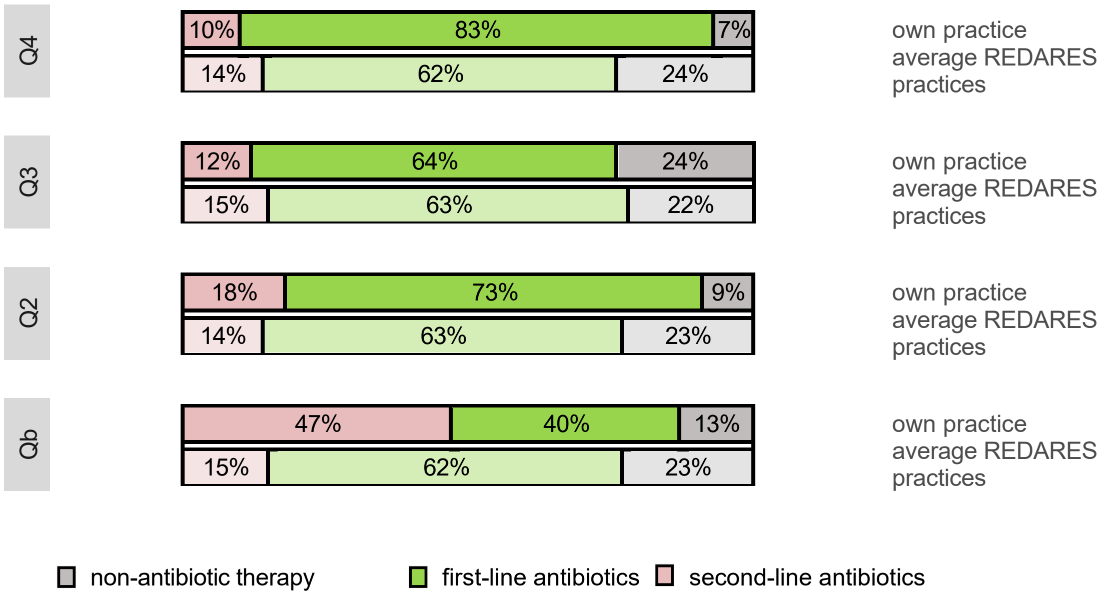

Supplementary Figure 2

Density plot of residuals from ANCOVA model for the primary endpoint

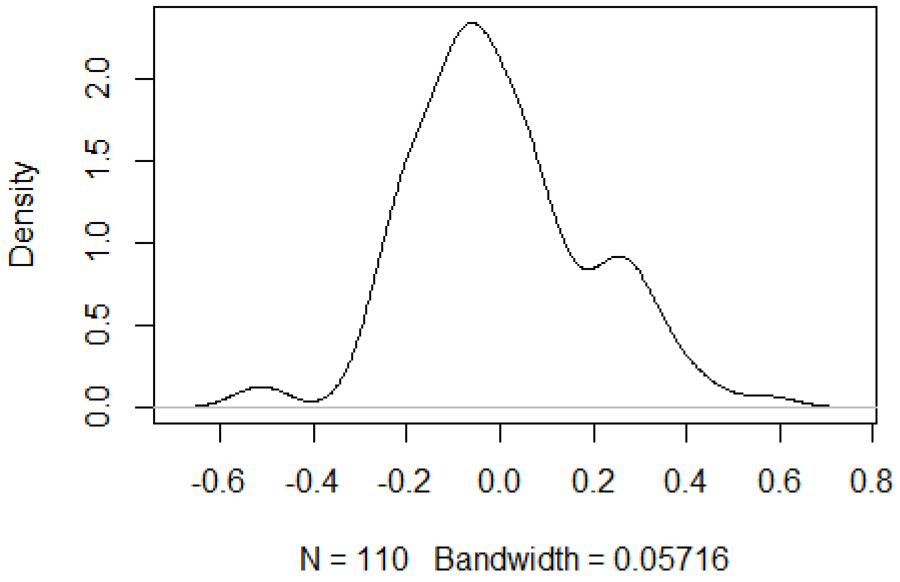

Supplementary material 1

Instructions for data extraction

Dear physician medical practice assistant,
These instructions serve as a reminder for the collection of cases in the RedAres study. If you have any questions, you can of course contact the study team at any time.
Please also note the training video on the RedAres homepage and the leaflet "Examples of case scenarios RedAres".

General:
Data collection for the RedAres study involves three steps:
1. case finding: identification of women with uncomplicated urinary tract infections in the practice management software (PMS).
2. data extraction into documentation folder: (by using case pages and transferring it into the tally sheet).
3. entry of aggregated data into the RedCap online database.

Detailed description of the individual steps:

- Search the practice management software (PMS) for cases of UTI and create a PMS results list
- Search for cases of patients diagnosed with UTI in the PMS and create a list of results, either at the end of the quarter or at shorter intervals.

Important: Always make surethat cases are not entered twice, always observe the date limits of the search when filtering. For practices with multiple doctors: Only include the cases of the GPs who have consented to RedAres.
The filter process depends on practice software and documentation habits in your practice.

Most frequent codes (ICD 10 codes for orientation):

- N 30.0 (Acute cystitis)
- N 30.8 (Other cystitis)
- N 30.9 (Cystitis, unspecified)
- N 39.0 (Urinary tract infection, site not specified)
- N 39.8 (Other specified disorders of urinary system)
- R 30.0 (Dysuria/Strangury)
- R 30.9 (Painful micturition, unspecified
- R 39.8 (Other and unspecified symptoms and signs involving the urinary system)

Case recording with the help of the extraction book:

- Enter data of each case from the PMS results list individually on the case pages (use a wipeable foil pen). The logical sequence of questions guides you through the case processing from the beginning to the end. When collecting data, please note possible free text fields and comments in the PMS
- Crosses are principally to be made on the case page. For the transfer to the tally sheet at the end, the tick in the outer (coloured) column is always decisive. Document the name of the antibiotic by hand if a different antibiotic has been prescribed only for the questions 7, 8 and 9.

- ALWAYS FILL IN THE CASE PAGES COMPLETELY FIRST! (only then transfer to the tally pages when it is certain that the case is included)
- On the "information sheet on inclusion/exclusion criteria" you will find an overview of cases that may not be included
- After completing the case pages, place them on the appropriate tally pages so that the outer column is exactly adjacent
- Based on what was ticked in the outer (coloured) column of the case pages, draw a tally mark on the tally sheet (use a waterproof pen). If a question is not marked in the outer column, do not draw a mark. For questions 7, 8 and 9, note the name of the antibiotic if "Other antibiotic" was ticked
- After transferring the results of all questions, tick off the case on the PVS results list, wipe off the marks on the case pages and start a new case
- It is best to use water to wipe the case, and alcohol-based disinfectant in between if necessary. When wiping the case sheets, it is best to take them off the tally sheet so that it does not get wet and smeared
- Please cross out cases that are not to be included on the PVS results list (e.g. if it turns out before or during data collection that it was not an uncomplicated UTI)
- In unclear cases, note this on the PVS results list and only process the case further or delete it from the list after consulting the doctor
- At the beginning of each new quarter, insert a new tally sheet and label it (e.g. 03/2021)

Important: Keep the old tally sheets even after the data transfer at the end of the quarter until the end of the study, as certain questions will only be counted at the end of the year

Counting and transferring the tally sheet to the data extraction sheet and entering it into the RedCap database (quarterly and once after one year)

- When all cases on the PVS results list have been gone through and the cases to be included have been recorded accordingly on the tally sheet, the tally sheet is counted and transferred to a data extraction sheet and to the RedCap database. The RedCap login data is in the envelope used for randomisation at the beginning of the study. For handling RedCap, please refer to the separate instructions for RedCap (screenshots)
- Please note: For data protection reasons, some questions are counted every quarter and some only at the end of the study period after one year; there are separate data extraction sheets and separate input masks in RedCap for each of these
- Counting quarterly: Questions 3-11 >> "Data extraction sheet for intervention practice".
- Count, transfer to the data extraction sheet and fax to the Institute of General Practice at the University Hospital of Wuerzburg or email.
- Open RedCap, enter the results of the counts in the corresponding fields
- Ignore questions 1, 2 and 12-17 now, they will be counted only in the end of the study
- Counting after one year: Questions 1., 2. and 12.-17. >> "Data extraction sheet for aggregated data after one year".
- The results for each question from all collected quarters have to be summed up.
- Transfer the respective sums to the data extraction sheet and fax it to the Institute of
- General Medicine at the University Hospital of Würzburg or email it
- Open RedCap, enter the totals in the corresponding fields.
-

Supplementary material 2

SOP data validation

Data validation 1st study quarter in intervention practices:

- Conduct data validation in each practice Schedule an appointment in the second or last third of the first study quarter with the medical practice assistant
- Extract 3-5 cases together with the medical practice assistant on site in order to verify the correct use of the "extraction book" going through the patients question by question together
- Select cases randomly so that they are not predictable for the medical practice assistant

Procedure in case of discrepancies (between EMR and extracted data):

- If discrepancies occur, check further (e.g. 5 cases again) and
- If necessary, in the case of further discrepancies, even complete manual checking

Data validation in control practices:

- If necessary, arrange a telephone appointment for data validation (or directly on site)
- Conduct data validation in 20-25% of all regional practices
- The validations process is the same as for intervention practices
